# Weekly Proactive Telephone Breastfeeding Standard Care by Lactation Consultants in the First Month Postpartum Prolongs Breastfeeding for Up to 6 Months

**DOI:** 10.3390/nu15092075

**Published:** 2023-04-25

**Authors:** Wei Qi Fan, Christopher Chan, Susan Paterson, Kathryn Foster, Michelle Morrow, Debra Bourne, Jodie Ashworth

**Affiliations:** 1 Northern Health, Melbourne, VIC 3076, Australia; 2 Faculty of Medicine, Dentistry and Health Sciences, The University of Melbourne, Melbourne, VIC 3010, Australia; 3 Butterfly Foundation, Palmwoods, QLD 4555, Australia

**Keywords:** exclusive breastfeeding, lactation consultant, telephone support

## Abstract

Many mothers, especially those with co-morbidities, do not achieve exclusive breastfeeding (EBF) for the first 6 months, with the loss of multiple health benefits including enhanced infant nutrition. We wished to evaluate whether proactive lactation consultant telephone advice in the first month postpartum improved breastfeeding rates for up to 6 months. A prospective cohort observational study was performed. Mother groupings included the following: Control (CG, *n* = 379)—standard postnatal care; Exposure (EG, *n* = 386)—standard postnatal care delivered by lactation consultant telephone contact for the first 3 weeks postpartum and then follow-up calls at 1, 3 and 6 months postpartum to ascertain breastfeeding status. Sore nipples (24%) and fussy/unsettled behaviour (14–19%) were common EG concerns. EG EBF rates were higher at 1 month (65% vs. 53%; *p* < 0.001), 3 months (57% vs. 49%; *p* = 0.041) and 6 months (45 vs. 33%; *p* < 0.001). EG EBF rates across the 6 months were higher for infants admitted to the NNU (52.9% vs. 37.5%, *p* = 0.003), obese mothers (58.3% vs. 37.2%, *p* < 0.001), mothers with depression (60.8% vs. 43.4%, *p* = 0.036) and all birth modes. Proactive early lactation advice significantly prolongs EBF and consequently enhances infant nutrition overall, including for mothers at risk of early breastfeeding cessation.

## 1. Introduction

Exclusive breastfeeding (EBF) is recommended in Australia until at least 6 months of age, conferring short- and long-term benefits in reduced morbidity risk and improved cognitive development to the infant and health benefits to the mother [1]. However, this is often not achieved, and mothers turn to artificial formula as ongoing breastfeeding is multifactorial, encompassing maternal psychosocial as well as practical concerns such as reassurance, positioning, newborn feeding behaviour and nipple pain [2].

While infant formula development is progressing, it is human milk that confers unique benefits. Important in this role is the lipid fraction of human milk, which differs markedly in both globule size and the complexity of lipid composition when compared with formula [3]. Research is continuing to reveal that certain lipid components are associated with both short- and longer-term benefits—such as specific long-chain fatty acids, which improve cognitive development, reduce asthma risk in school-age children [4] and protect against non-communicable diseases [5].

Despite EBF being clearly beneficial to an infant’s growth and development, there are a number of risk factors militating against EBF continuation. A study in the region served by our hospital—a multicultural, lower-socioeconomic growth corridor of a major Australian city—showed that while an almost 100% breastfeeding initiation rate was achieved, at 8 weeks postpartum, only 33% of obese mothers were still EBF compared with a 70% EBF rate amongst mothers with a normal Body Mass Index (BMI) [6]. Caesarean delivery increases the risk of not initiating breastfeeding as well as decreasing EBF rates [7]. Premature infants admitted to a neonatal intensive care unit (NICU) have low EBF rates at 6 months [8], and their mothers have fewer positive interactions and less attachment [9]. Maternal depression is another risk factor. While the presence of depression does not seem to influence breastfeeding initiation, mothers who have persistent depressive symptoms are more likely to breastfeed for less than 1 month [10]. 

Two common strategies for providing EBF support are home visits and telephone calls. Trained health workers visiting mothers at home during the first 5 weeks postpartum resulted in a 14% lower breastfeeding cessation at 6 months [11]. Telephone support is an attractive option for hospitals, as the cost is less than home visiting. The quality of a call is important. In calls made by qualified nurses to mothers with breastfeeding concerns, ineffective calls have been identified as those focusing on technicalities; undermining calls made moral judgements of a mother’s behaviour, and supportive calls were personalised, respectful and sharing [12]. The effectiveness of telephone-based breastfeeding support has been mixed. In the case of premature infants after NICU discharge, proactive telephone support did not result in improved breastfeeding rates 8 weeks later [9]. Similarly, a pilot study of telephone support for mothers living in a disadvantaged area did not show improved breastfeeding rates [13], nor did a Spanish study of mothers with term infants show improved breastfeeding rates at 6 months—possibly due to parenting, social and cultural factors [14]. However, telephone support of obese mothers did prolong breastfeeding at 6 months [15], and an Australian study of proactive peer (mother-to-mother) telephone support also showed improved breastfeeding rates at 6 months [16]. A Cochrane Review concluded that, in general, breastfeeding support improves the duration of EBF when the support is offered by trained staff, is scheduled, suits the needs of the local population and is more effective if breastfeeding initiation rates are high [17].

Due to the active promotion of breastfeeding in our region and breastfeeding initiation being high [6], we hypothesized that exposing newly breastfeeding mothers to proactive, weekly lactation consultant telephone-based standard care would increase breastfeeding duration compared with the current situation where mothers must actively seek out the same standard post-discharge breastfeeding care available in the community. Consequently, we designed and carried out a prospective cohort observational study to observe breastfeeding rates at 1, 3 and 6 months of age and to evaluate breastfeeding rates against concerns specific to our region such as obesity, maternal depression and infants admitted to our Neonatal Unit (NNU). 

## 2. Materials and Methods

### 2.1. Study Design and Participants

This was a single-site, prospective cohort observational study with the aim of observing whether a lactation consultant-led, telephone-based support program in the first month postpartum improved breastfeeding rates for up to 6 months postpartum both generally (primary outcome) and in mother–infant pairs at risk of early breastfeeding cessation (secondary outcome). This study was solely designed to refine measures to improve access to the standard care of postnatal breastfeeding support. 

The study was conducted at The Northern Hospital (TNH), a major, 740-bed community hospital north of Melbourne, Australia. TNH serves both the northern suburbs and country areas of Victoria. There are approximately 4000 births annually. The TNH NNU is an 18-bed high-level nursery that cares for infants from 31 weeks gestation. 

Mothers with healthy infants who had commenced breastfeeding upon postnatal discharge from TNH and were English-speaking were eligible for inclusion. Exclusion criteria included illnesses or medications that are contraindicated with breastfeeding or may significantly compromise the success of breastfeeding and mothers at less than 36 weeks gestation at time of delivery. Mothers in the postnatal ward were identified and screened for eligibility. Potential participants were given a copy of a plain-language statement that outlined the purpose of the study, gave an overview and informed them of risks and benefits and the protection of recorded personal information. Participants were given the opportunity to discuss any concerns with the investigators or lactation consultants. Individual informed consent was obtained. Those who consented were assigned to either Exposure or Control Groups. We chose to use simple randomisation for assignments rather than assign sequentially to aid in compensating for confounders such as maternal attitude to breastfeeding, bias towards the lactation consultants, socioeconomic factors and factors known to inhibit breastfeeding such as obesity.

### 2.2. Control and Exposure Groups

The Control Group received current, established counselling and postnatal and post-discharge care as provided to all mothers. This included inpatient lactation support with midwives and lactation consultants. Outpatient breastfeeding standard care if accessed by a mother is available through maternal child health services and community breastfeeding clinics, specialist breastfeeding services and general practitioner follow-ups. For example, the Australian Breastfeeding Association provides a 24-h telephone helpline and live-chat service as well as a Maternal Child Health Nurse 24-h hotline. The Exposure Group received proactive outpatient breastfeeding standard care via telephone contact weekly in the first 3 weeks following birth. All calls were made by TNH’s International Board-Certified Lactation Consultants (International Board of Lactation Consultant Examiners, Fairfax, VA 22030, USA) (IBCLC) and followed a structured questionnaire. This questionnaire formed the basis of all recorded data sheets (see Supplementary Materials). The development of the questionnaire/data collection sheet was a collaboration between the researchers—neonatologists and lactation consultants. In reviewing the literature relating to telephone breastfeeding support, there were 2 opposing approaches—either very structured questionnaires based on well-researched evidence such as maternal breastfeeding scales [9] or a desire to emphasise the quality of the relationship between lactation support personnel and breastfeeding mothers, which lacked a formal questionnaire and drew on the expertise of the lactation consultant [12]. We chose to take an approach between these 2 extremes with questions based on professional lactation consultant experience [18] but structured in such a way as to promote a collaborative relationship between the lactation consultant and mother. During these phone calls, the lactation consultant enquired about areas of concern, providing standard breastfeeding care. Feeding status was also recorded. Mothers in both groups then received follow-up telephone contact at 1, 3 and 6 months to assess breastfeeding status. Telephone contact was discontinued when mothers were no longer expressing breastmilk or breastfeeding their babies. If there were any health concerns for either the mother or baby during these phone calls, appropriate follow-up advice was given.

### 2.3. Data Collection and Analysis

The sample size for the study was calculated using the OpenEpi program [19] with a baseline 6-month exclusive breastfeeding rate of 29%, as per the Australian Bureau of Statistics [20]. Assuming an improvement of 10%, 696 participants were required (alpha = 0.05, beta = 0.2, power = 0.8). When taking into consideration the possible 10% lost during follow-up, we aimed to recruit 765 participants. Due to funding restrictions on research staffing, sequential recruitment of participants was not possible. Recruitment was via convenience sampling, with the majority of recruitment performed by the lactation consultants on their routine rostered sessions from Monday to Friday, with some additional recruitment performed by midwives. Retrospective demographic data were accessed from the TNH Birthing Outcomes Summary and Medical Records. Demographic data collected included (for the mother) date of admission, date of discharge, weight, height, BMI (obesity BMI (≥30)), maternal complications, gravida, parity and maternal depression as identified by the Edinburgh Postnatal Depression Scale (completed at the antenatal clinic booking-in visit) and (for the infant) sex, date of birth, date of discharge, days admitted to NNU (if required), gestation, mode of birth, birth weight and neonatal complications. All additional data were collected via the patient questionnaire. Data from the standard questionnaire and Medical Records were transferred to Microsoft Excel for analysis. Patient data were recorded as missing if participants were unable to be contacted despite multiple attempts to call. In evaluating breastfeeding status, “exclusive breastfeeding” follows the WHO definition: “the infant receives only breast milk. No other liquids or solids are given—not even water—with the exception of oral rehydration solution, or drops/syrups of vitamins, minerals or medicines” [21].

The lactation consultants were unblinded to participant groups when making telephone calls. Due to the Exposure, participants in both the Control and Exposure Groups would have become aware of their group status. Study questionnaires and data entry were blinded to the investigator until all data were entered. The data were collated and analysed using Microsoft Excel and the NCSS 12 statistical program (NCSS, St. Albans, UT, USA). Logistic regression was used to calculate the odds ratio (OR) and OR 95% confidence intervals. Chi-square analysis was used to determine *p*-values. All results were interpreted using *p* < 0.05 as statistically significant.

### 2.4. Ethics Approval

This study was granted ethics approval by the Austin Health Human Research Ethics Committee, which operates in accordance with the Australian National Health and Medical Research Council’s National Statement on Ethical Conduct in Human Research. HREC Reference number: LNR/17/Austin/371. Although not a clinical trial as defined by the US NIH, as the study was designed to solely finetune existing standard care [22], the study was registered as follows: Australian New Zealand Clinical Trials Registry (ANZCTR) Australian clinical trials registration number: ACTRN12618001225202 (registered 20 July 2018).

## 3. Results

Throughout this report, we have used the term Breast Milk Feeding (BMF), which equates to EBF as defined above, as at some time points, infants were fed expressed breastmilk via tube or bottle rather than on the breast.

### 3.1. Participation

**Figure 1:**
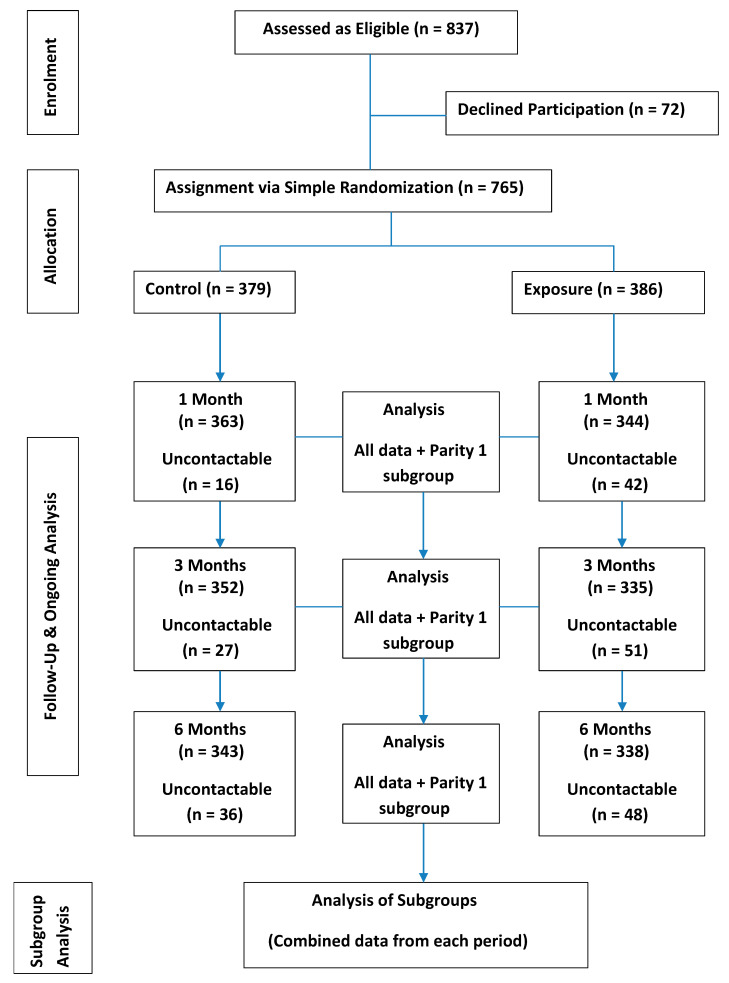
Participant Flow Diagram.

The study was commenced in February 2018 and completed in March 2020. A total of 765 breastfeeding mothers were enrolled in the study (eligible 837, declined 72). There were 379 participants in the Control Group and 386 in the Exposure Group. All mothers in the Exposure Group were contacted via phone and proactively offered standard care breastfeeding support during the Exposure period of 3 weeks following birth (Figure 1). Control group mothers could access breastfeeding standard care at their own discretion through multiple community options. At 1, 3 and 6 months, mothers from both the Control Group and Exposure Group were contacted to ascertain feeding type. Once breastfeeding ceased, these mothers were not further followed up.

### 3.2. Baseline Characteristics—Table 1

There were no statistical differences between the Control and Exposure cohort comparisons, except for an increase in primiparous mothers in the Exposure Group. While twins were not excluded from the study, there was only one twin set enrolled (Control Group).

### 3.3. Feeding Outcomes

At 1, 3 and 6 months (Table 2), the percentage of BMF mothers was significantly higher in the Exposure Group. There was less artificial feeding (AF) at 6 months but not at 1 or 3 months for the Exposure Group. At 1 and 6 months, the number of infants having mixed feeding (MF) was significantly lower.

### 3.4. Exposure Group Well-Being and Concerns

The most common concerns raised during the 3 weeks of Exposure support (Table 3) were sore nipples, fussy/unsettled infant behaviour and feed frequency. The most common management advice given across the 3 weeks was regarding infant feeding position and attachment to the nipple.

### 3.5. Primiparous Feeding Outcomes

To address the bias of more primiparous mothers in the Exposure Group, Table 4 details the feeding type outcomes for primiparous mothers only. At 1 month for AF and at 1 and 3 months for MF, there were significantly fewer primiparous infants in the Exposure Group. At 1, 3 and 6 months, there were significantly more BMF infants in the Exposure Group.

### 3.6. Feeding Outcomes for Mothers at Higher Risk of Early EBF Cessation

**Figure 2:**
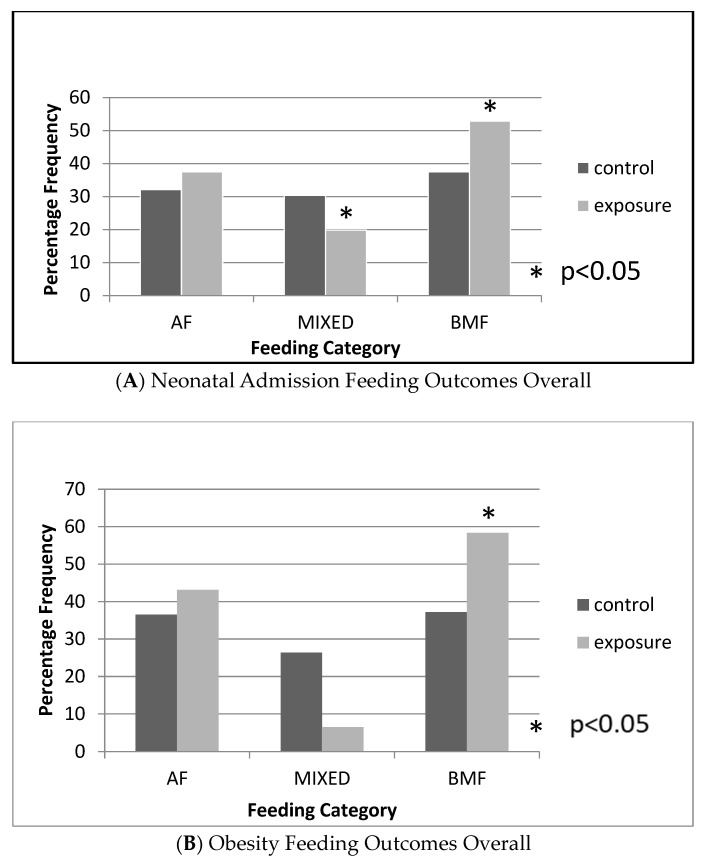

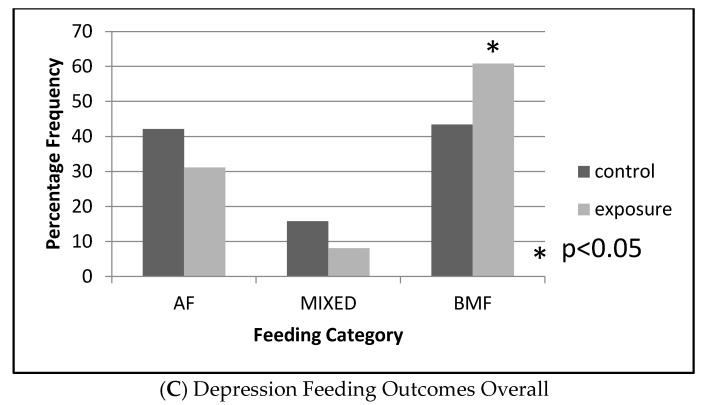
Overall Feeding Outcomes for Mothers at Higher Risk of Early BF Cessation. Total number of data points across the 6 months (Exposure:control): (**A**) (187:184); (**B**) (123:148); (**C**) (74:76). EBF rates across the 6 months for infants admitted to the NNU: 53% vs. 38%, *p* = 0.003. EBF rates across the 6 months for obese mothers: 58% vs. 37%, *p* < 0.001. EBF rates across the 6 months for mothers with depression: 61% vs. 43%, *p* = 0.036. MIXED rates across the 6 months for infants admitted to the NNU: 30% vs. 18%, *p* = 0.022. MIXED rates across the 6 months for obese mothers: 18% vs. 3%, *p* = 0.040. All the above comparisons are for the Control Group and Exposure Group.

**Figure 3:**
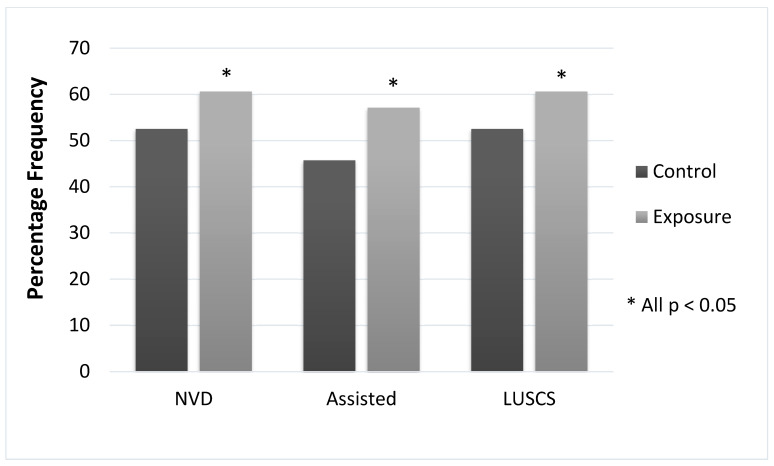
BMF Compared for Birth Mode. Data are cumulative for the 6 months of the study. NVD—normal vaginal delivery. Assisted—assisted vaginal delivery. LUSCS—caesarean delivery. NVD, *p* = 0.020; assisted, *p* = 0.044; LUSCS, *p* < 0.0001.

Whether the telephone breastfeeding support program would be helpful to mothers in categories with a known risk of earlier breastfeeding cessation and whether birth mode impacted support program outcomes were of interest. The data presented in Figure 2 and Figure 3 are for the entire study—all data points at 1, 3 and 6 months were pooled to give an overall view of the three higher risk categories of NNU admission, maternal obesity, maternal depression and birth mode. Over the course of the study, infants who were admitted to the NNU and infants of mothers with obesity and depression had significantly higher BMF rates for the Exposure Group compared with the Control Group. MF rates were significantly lower for infants admitted to NNU and for infants of obese mothers. There was no difference in the rates of AF for any of these three higher-risk categories. Regardless of birth mode, BMF rates were significantly higher for the Exposure Group.

## 4. Discussion

This prospective cohort observational study observed that a telephone-based EBF support program, managed by two qualified IBCLCs during the first month after birth, was effective in increasing the duration of EBF for up to 6 months.

A recent telephone-based study of breastfeeding support across three other hospitals in Melbourne reported slightly higher (but not statistically significant) EBF rates at 6 months [16]. In this study, rather than IBCLCs, peer-to-peer support was offered by mothers who had previously breastfed for at least 6 months. Although both studies were in the same city, were telephone-based and had similar socioeconomic demographics, there were key differences such as the qualification of supporters; the length of active support; and the mother and infant selection criteria. A 2017 Cochrane Review of breastfeeding support for healthy mothers and term babies highlighted the difficulties of drawing inter-study conclusions [17]. The review linked the heterogeneity of findings with factors such as setting, the population groups studied, the level of standard care available, the type and timing of measured outcomes and biasing due to practical difficulties with blinding. Consequently, the findings of this study, while relevant to our setting and population, may need to be used with caution in applying them more generally.

Table 2 presents the feeding outcomes over the course of the study. While it is encouraging that the Exposure Group breastfeeding rate is significantly higher than the Control at 1, 3 and 6 months, there is room for improvement. At 1 month, the Exposure Group BMF rate is higher than the Control (73% vs. 55%), but then, there is a large percentage drop at 3 months by 15 percentage units. As MF remains the same and AF increases, it appears that almost all these Exposure Group mothers switched from breastfeeding to AF at 3 months. Such a rapid drop off in breastfeeding between 1 and 3 months did not occur in a study where ongoing peer-to-peer telephone support calls were made [16] or in a lactation-consultant-led study where text and phone calls were made on a weekly basis until 6 months were up [23]. The Cochrane Review [17] did not draw any conclusions regarding the duration of telephone support but did conclude that the duration of face-to-face support was an important factor in exclusive breastfeeding. Consequently, our study results suggest that the identification and nurturing of mothers likely to switch to AF in the first few months would be beneficial in improving longer-term breastfeeding rates.

It is encouraging for the potential of ongoing breastfeeding that a high percentage of mothers reported comfortable breastfeeding during the Exposure period. However, above 40% of mothers still had concerns at 3 weeks, suggesting that extending telephone support further would have aided in promoting ongoing breastfeeding. The concerns raised by mothers are similar to those reported previously [2,24,25]. One study was also conducted in Melbourne, and as in ours, the more frequent issues raised were supply and demand, positioning and attachment and feed frequency [2]. Issues raised by mothers and managed by our IBCLCs, such as painful nipples, milk supply/milk expression and attachment and positioning, have been shown to be associated with early breastfeeding cessation [26].

An effective breastfeeding support program needs to consider parity, as the concerns and breastfeeding issues of first-time mothers differ from multiparous mothers. A 2015 randomised controlled study of over 1000 mothers reported that primiparous mothers took longer to attempt breastfeeding; had fewer attempts at breastfeeding in the first 24 h; had more early breastfeeding problems; and were more likely to be discharged with MF. Multiparous mothers were more likely to breastfeed until 6 months and had a lower hazard of breastfeeding cessation [27]. Our study had a higher frequency of primiparous mothers in the Exposure Group (Table 1). In order to compensate for this bias, primiparous mothers were compared directly (Table 4). Our IBCLC-led, telephone-based support program had a significantly positive impact on primiparous mothers, with higher rates of BMF at 1, 3 and 6 months in the Exposure Group, while at 1 month, AF rates were lower, and at 1 and 3 months, MF rates were also lower in the Exposure Group.

The admission of an infant to a NICU, maternal obesity and maternal depression are risk factors for the early cessation of breastfeeding [6,8,10]. Preterm infants have weakened suck and immature development, which presents obstacles to breastfeeding both to the baby and mother, as the expression of breastmilk is necessary and feeding is often via tube or bottle [8]—increasing the risk of early breastfeeding cessation. In our study, all infants were 36 weeks gestation or older when admitted to the NNU and so did not necessarily have the concerns of preterm infants. There appears to be a lack of information in the literature regarding how an NNU admission of near (or late preterm) and full-term infants with morbidities, such as respiratory concerns or infection management, can impact breastfeeding and maternal bonding. Our study (Figure 2A) showed that over the course of the 6-month study period, infants admitted to the NNU benefited from the telephone support program, with higher BMF rates and lower MF.

Obesity in mothers impacts breastfeeding with both psychological and biological factors. One factor is a lower prolactin response to infant suckling, which may compromise milk production and lead to an earlier cessation of breastfeeding [28]. Women who have large breasts may have difficulties with infant attachment [29]. Additionally, psychosocial factors such as intention to breastfeed, schooling and self-efficacy may impact success [30]. One study reported that telephone-based breastfeeding support prolonged both exclusive and partial breastfeeding up to 6 months postpartum [15]—results that are not dissimilar to this study, which showed that such support increased BMF while decreasing the rate of MF (Figure 2B).

Maternal anxiety and depression are associated with early breastfeeding cessation, and ironically, breastfeeding cessation impacts maternal anxiety and depression symptoms [31]. Our study (Figure 2C) showed that, for mothers with depression, telephone breastfeeding support improved breastfeeding rates. This finding is positive, as breastfeeding itself may help reduce the symptoms of depression up to 3 months postpartum [32].

As detailed in Figure 3, across the study period, Exposure Group BMF rates were significantly higher for each birth mode. Especially relevant is that Exposure Group mothers delivering via caesarean had higher BMF rates than the Control Group, as a caesarean is considered to be major abdominal surgery associated with negative maternal well-being and interferes with the physiology of lactation in the early postpartum period [7].

As previously mentioned, our results need to be interpreted in context. In general, telephone support studies have inherent liabilities and limitations—the effectiveness of voice alone is limited, as visual aids such as models, hand movements and photographs are not possible with the resultant potential for miscommunication and misunderstandings (raising the possibility of an improved support program via video). Due to the restriction of using the English language only in this study, capturing a representative sample of mother–infant pairs in our multi-ethnic community could not be guaranteed. Another limitation is that potential confounders such as maternal ethnicity and socioeconomic data, including profession and education level, were not assessed. Maternal ethnicity and sociodemographic evaluation are clearly pertinent to developing a proactive breastfeeding support program in our community, but due to funding shortfalls for language interpreters and the clinical research nursing staff, these factors were not assessed in this study. However, the positive results of this study will hopefully be an incentive for funding a more comprehensive study. Another limitation is that of an objective paediatric assessment during the period of the study. Such assessments are carried out at regular intervals during an infant’s first year but at the community level. Due to privacy reasons, these assessments were not accessible to the researchers. A strength of the study was that it had a competitive number of participants compared with similar published studies [15,16,33]. Due to the nature of the study, blinding was not possible. Limiting the number of support IBCLCs to two provided continuity and familiarity for the mothers over the course of the study and enhanced the potential of supportive calls [12].

Given the caveat mentioned above concerning the difficulties in translating the findings of this study to other regions and localities, our observation that breastfeeding duration can be prolonged by a simple and cost-effective method of the proactive exposure of newly breastfeeding mothers to standard care is a significant health finding. Human breast milk is unique, and the benefits accrue both to the mother (such as reducing symptoms of depression) and the infant. Particularly relevant is our finding that breastfeeding is prolonged for obese mothers, confirming a similar finding in a previous study of telephone support [15]. Obesity and overweightness are significant noncommunicable diseases in our community, with multiple poor health outcomes and a maternal–child overweightness/obesity linkage [34]. Breastfeeding itself has the potential to break this linkage, as it has been shown to reduce obesity in an infant’s later life. Conversely, formula-fed infants have higher rates of obesity in later life compared with those who are breastfed [5]. Breastfed infants, compared with formula-fed infants, have a different pattern of adiposity, and this effect has been associated with the unique composition of the human milk lipid fraction. There are a number of potential mechanisms, including breastfed babies having increased levels of protective thermogenic beige adipose tissue, which is sustained by a specific lipid present in breastmilk [5].

## 5. Conclusions

This study has shown that for a large community hospital, the cost-efficient alternative of a telephone-based breastfeeding support strategy providing proactive standard care operated by dedicated lactation consultants can have a positive impact on breastfeeding rates up to 6 months postpartum. Importantly, this support was associated with improved breastfeeding outcomes in maternal groupings at risk of early breastfeeding cessation—primiparous mothers; maternal obesity and depression; caesarean delivery; and infants requiring admission to NNU. The study has also highlighted the potential for improved future initiatives: the targeting of mothers assessed as likely to cease breastfeeding early and for video consultation to provide a more balanced supportive program by allowing visual communication.

## Figures and Tables

**Table 1:** Participants’ Baseline Characteristics.

Characteristics	*Descriptor*	Control Group *n* = 379	Exposure Group *n* = 386	*p*-Values
**Mother’s age (mean yrs ± s.d.)**		30.3 ± 5.0	29.8 ± 5.1	0.112
**Parity status (*n*, %)**	Primiparous	167 (44.0%)	221 (57.2%)	<0.001
**GBS status (*n*, %)**	Positive	78 (20.6%)	80 (20.7%)	0.960
**Meconium-stained liquor (*n*, %)**	Present	58 (15.3%)	58 (15.0%)	0.915
**Type of delivery (*n*, %)**	Normal vaginal	156 (41%)	159 (41%)	0.189
	Assisted vaginal	58 (15%)	61 (16%)	
	Elective LUSCS	61 (16%)	43 (11%)	
	Emergency LUSCS	102 (27%)	121 (31%)	
**Gestation (mean wks ± s.d.)**		39.0 ± 1.2	39.0 ± 1.3	0.599
**Infant birth weight (mean g ± s.d)**		3362 ± 479	3303 ± 481	0.090
**Infant gender (*n*, %)**	Female	178 (47%)	205 (54%)	0.076
**APGAR scores (mean ± s.d)**	1 min	8.5 ± 1.2	8.5 ± 1.3	0.655
	5 min	9.0 ± 0.5	8.9 ± 0.6	0.435
**First feed mode (*n*, %)**	Breast	247 (66%)	233 (61%)	0.259
	Breast and formula	7 (2%)	14 (4%)	
	Breast and EBM	62 (16%)	66 (17%)	
	EBM	47 (12%)	49 (15%	
	Formula feed	14 (4%)	9 (2%)	
**Initial skin contact duration (minutes)**	Median (IQR)	60 (60)	60 (60)	0.626

s.d.—standard deviation, yrs—years, wks—weeks.

**Table 2:** Feeding Outcomes.

Feeding Type Per Interval	Control Group *n* = 379	Exposure Group *n* = 386	OR (95% CI)	*p*-Values
**BMF (*n*, %)**				
1 month	201 (55%)	251 (73%)	0.46 (0.34–0.63)	<0.001
3 months	174 (49%)	195 (58%)	0.70 (0.52–0.95)	0.022
6 months	123 (36%)	175 (52%)	0.52 (0.38–0.79)	<0.001
**MIXED (*n*, %)**				
1 month	107 (29%)	56 (16%)	2.19 (1.45–3.09)	<0.001
3 months	77 (22%)	56 (17%)	1.40 (0.94–2.04)	0.101
6 months	72 (21%)	42 (12%)	1.87 (1.24–2.67)	0.003
**AF (*n*, %)**				
1 month	55 (15%)	37 (11%)	1.48 (0.95–2.31)	0.094
3 months	101 (29%)	84 (25%)	1.20 (0.86–1.69)	0.303
6 months	149 (43%)	121 (36%)	1.38 (1.01–1.87)	0.042

At 1 month, Control, *n* = 363; Exposure, *n* = 344. At 2 months, Control, *n* = 352; Exposure, *n* = 335. At 3 months, Control, *n* = 343; Exposure, *n* = 338.

**Table 3:** Exposure Group Well-Being and Concerns.

Concerns and Management	Week One	Week Two	Week Three (95% CI)
Breastfeeding is comfortable	66%	70%	75%
Mothers with any concerns	55%	43%	44%
Most common concerns	Sore nipples (24%) Frequency of feedings (17%) Milk collection and storage (16%) Fussy/unsettled baby (13%)	Fussy/unsettled baby (14%) Frequency of feeds (13%) Sore nipples (11%) Low supply (11%)	Fussy/unsettled baby (19%) Frequency of feeds (14%) Low supply (9%) Sore nipples (8%)
Advice and management plan	Discussion about position and attachment (40%) Expressing breastmilk pump (32%) Milk collection and storage (19%) Management of sore nipples (17%)	Discussion about position and attachment (25%) Expressing breastmilk pump (22%) Management of fussy/unsettled baby (9%) Management of low supply (8%)	Discussion about position and attachment (20%) Expressing breastmilk pump (16%) Management of fussy/unsettled baby (10%) Management of low supply (8%)

**Table 4:** Primiparous Feeding Outcomes.

Feeding Type Per Interval	Control Group	Exposure Group	OR (95% CI) ()95%CI)	*p*-Values
**BMF (*n*, %)**				
1 month	73 (45.3%)	138 (70.1%)	0.35 (0.23–0.55)	<0.001
3 months	66 (42.6%)	110 (55.6%)	0.59 (0.39–0.91)	0.018
6 months	50 (32.1%)	94 (47.7%)	0.52 (0.33–0.80)	0.003
**MIXED (*n*, %)**				
1 month	55 (34.2%)	36 (18.3%)	2.32 (1.43–3.77)	0.001
3 months	39 (25.2%)	29 (14.6%)	1.96 (1.95–3.35)	0.015
6 months	31 (19.9%)	28 (14.2%)	1.50 (0.85–2.62)	0.196
**AF (*n*, %)**				
1 month	33 (20.5%)	23 (11.7%)	1.95 (1.09–3.40)	0.028
3 months	50 (32.3%)	59 (29.8%)	1.12 (0.71–1.77)	0.644
6 months	75 (48.1%)	78 (39.6%)	1.41 (0.92–2.16)	0.130

At 1 month, Control, *n* = 161; Exposure, *n* = 197. At 3 months, Control, *n* = 155; Exposure *n* = 198. At 6 months, Control, *n* = 156; Exposure, *n* = 197.

## Data Availability

The data sets generated and/or analysed are available from the corresponding author upon reasonable request.

## Supplementary Materials

The following supporting information can be downloaded at https://www.mdpi.com/article/10.3390/nu15092075/s1: Study Questionnaire.
