# Supplementary material for: Weekly Proactive Telephone Breastfeeding Standard Care by Lactation Consultants in the First Month Postpartum Prolongs Breastfeeding for Up to 6 Months
Source: Nutrients. 2023 Apr 25;15(9):2075. doi: 10.3390/nu15092075 (PMC10180720; doi:10.3390/nu15092075)
Supplement: Supplementary file 1 [file nutrients-15-02075-s001.zip › nutrients-2343230-supplementary.pdf]

Data Collection Sheet for Telephone Calls for both exposure and control groups

Before 4 weeks (exposure group), then at 4 weeks, 3 months and 6 months (both groups)

1. Date of Call __/__/20__ S M T W T F S S
2. Time ___________ AM PM UNKNOWN
3. Mothers name_____________________ DOB / / Address ________________________________
4. Date / / time returned________ UR number_____________________________
5. Phone number:____________________ Age of baby:____________________________
6. **INTRODUCTORY QUESTIONS:**
   a. Is it convenient for us to chat now? ☐ yes ☐ no
   b. Is breast feeding comfortable? ☐ yes ☐ no
   c. Are you having fun with your baby? ☐ yes ☐ no
   Is baby smiling ☐ yes ☐ no, having good wet nappies ☐ yes ☐ no, having good bowel motions / passing wind ☐ yes ☐ no?
   d. Is baby waking spontaneously to feed and look around? ☐ yes ☐ no
   e. At the moment is your breastfeeding experience positive ☐, neutral ☐ or negative ☐?

   [If these questions are answered affirmatively, then breastfeeding is most likely satisfactory.]

7. **FOLLOW UP QUESTIONS** (where applicable):
   a. Are you in pain when breastfeeding? ☐ yes ☐ no
   b. How are your support networks going eg partner, family, friends? ☐ satisfied ☐ not sure
   c. Do you feel comfortable breastfeeding around others or in public? ☐ yes ☐ no
   d. How many times a day do you breast feed? ☐ yes ☐ no
   e. How long does each breastfeeding session last on average? _____minutes
   f. Is you baby gaining weight? ☐ yes ☐ no
   g. How long do you intend to continue breastfeeding? _____months

8. **Concerns from the mother**: (Tick one category for each issue discussed)

☐ Sore nipples/bleeding nipples	☐ Pump hire/equipment questions	☐ Medication for mum
☐ Medication for baby	☐ Formula	☐ Fussy /unsettled baby
☐ Tongue Tie/Lip tie	☐ Maternal diet	☐ Sleepy baby
☐ Mastitis	☐ Oversupply	☐ Low supply
☐ Milk collection and storage	☐ Engorged	☐ Frequency of feeds

Management Plan (Tick one category for each issue discussed)

☐ Discussion about positioning and attachment	☐ Bottle feeding/teat	☐ Hand expressing
☐Transitioning bottle to breast	☐ Management of sore nipples	☐ Management of weight loss
☐ Expressing breastmilk pump	☐ Management of low supply	☐ Management of mastitis
☐ Management of breast fullness	☐ Management of thrush	☐ How to use nipple shield
☐ Management of engorgement	☐ Management of sleepy baby	☐ Milk collection and storage

Comments:

____

____

____

____

How are you feeding your baby?

☐Fully breastfeeding ☐Breastfeeding/EBM ☐Breastfeeding/EBM/AF

Outcome of Call

☐Advice given ☐Advised to see MCHN ☐ Referral to Northern BF clinic.

☐Advised to see GP ☐ Referral to Paediatric team at TNH ☐Unable to reach caller ☐ Other

NOTES ON DATA COLLECTION.

The aim of this prospective study is to observe if the duration of exclusive and partial breastfeeding is prolonged. The questions are designed to reveal the physical and psychological aspects related to breastfeeding and the well-being of the mother and child.

The *introductory* set of questions are designed to get an overall picture of whether breast feeding is progressing satisfactorily.

Following this is a group of *follow up questions*. These questions are given as a guide to following up concerns raised by answers to the introductory set.

Finally, there is a section listing common concerns and management outcomes which need to be ticked.
